# High-Throughput Prediction of Protein–Protein Interactions Uncovers Hidden Molecular Networks in Biosynthetic Gene Clusters

**DOI:** 10.34133/csbj.0149

**Published:** 2026-06-29

**Authors:** Yoshitaka Moriwaki, Taro Shiraishi, Yohei Katsuyama, Kenichi Matsuda, Jie Zhang, Toyoyuki Ose, Atsushi Minami, Hideaki Oikawa, Tomohisa Kuzuyama, Ryuichiro Ishitani, Tohru Terada

**Affiliations:** ^1^ Department of Computational Drug Discovery and Design, Medical Research Laboratory, Institute of Integrated Research, Institute of Science Tokyo, Tokyo 113-8510, Japan.; ^2^ Department of Biotechnology, Graduate School of Agricultural and Life Sciences, The University of Tokyo, Tokyo 113-8657, Japan.; ^3^ Department of Chemistry, Graduate School of Science, Kyoto University, Kyoto 606-8502, Japan.; ^4^ Collaborative Research Institute for Innovative Microbiology, The University of Tokyo, Tokyo 113-8657, Japan.; ^5^ Faculty of Pharmaceutical Sciences, Hokkaido University, Sapporo 060-0812, Japan.; ^6^ Faculty of Advanced Life Science, Hokkaido University, Sapporo 060-0810, Japan.; ^7^ Department of Chemistry, Institute of Science Tokyo, Tokyo 152-8551, Japan.; ^8^ Division of Chemistry, Faculty of Science, Hokkaido University, Sapporo 060-0810, Japan.

## Abstract

Biosynthetic gene clusters (BGCs) are contiguous genomic regions that encode diverse proteins responsible for natural product biosynthesis. These proteins collectively produce various secondary metabolites with complex chemical structure, including antibiotics and mycotoxins, yet the complete biosynthetic pathways have been experimentally elucidated for only a limited number of compounds. Recently, protein–protein interactions within BGCs have been recognized as key determinants of intermediate transfer, enzymatic regulation, and structural stability. However, many BGCs still contain proteins of unknown function that cannot be predicted by conventional sequence-based bioinformatics tools, hindering a comprehensive understanding of their biosynthetic pathways. To address this challenge, we built a high-throughput complex prediction pipeline by replacing AlphaFold3’s multiple sequence alignment generation with a faster MMSeqs2. We systematically screened 487,828 protein pairs derived from 2,437 BGCs registered in the Minimum Information about a Biosynthetic Gene cluster database and predicted 15,438 heteromeric interactions with an interface predicted TM score ≥ 0.6. Among them, 1,390 protein pairs exhibited structural homology with a root mean square deviation ≤ 2.0 Å. Our analysis demonstrates that AF3-based complex prediction matches experimental results with high confidence for most proteins and reveals many uncharacterized but novel heterocomplexes within BGCs. These findings will facilitate experimental verification of unidentified enzymatic reactions leading to the final products.

## Introduction

Secondary metabolites are natural products produced by organisms that are not directly involved in their growth, development, or reproduction. Unlike primary metabolites, such as amino acids, nucleotides, and carbohydrates, secondary metabolites often play specialized roles in ecological interactions—such as defense against predators [1], competition with other organisms [2], or symbiotic relationships [3]. These compounds are typically produced by biosynthetic gene clusters (BGCs), which are colocated sets of genes that collectively encode enzymes required to produce a specific metabolite and auxiliary proteins such as transporter and regulatory proteins [4,5]. Secondary metabolites contain a wide variety of chemical structures and classes, including nonribosomal peptides, polyketides, ribosomally synthesized and posttranslationally modified peptides, terpenes, and saccharides, which have attracted substantial interest from both enzymology and organic chemistry. Since the 2000s, progress in sequencing technologies, such as next-generation sequencing and metagenomic analysis, along with the development of bioinformatics tools and the expansion of genomic databases, has advanced research on secondary metabolites derived from uncultivable microorganisms. For example, antiSMASH [6–13] is a widely used bioinformatics tool for the identification, annotation, and analysis of BGCs in microbial genomes. It also classifies clusters into known types by comparing them to curated reference datasets, including the Minimum Information about a Biosynthetic Gene cluster (MIBiG) database [14]. DeepBGC [15] incorporates deep learning and natural language processing strategies to capture genomic positional dependencies and order information that are not considered in hidden Markov model (HMM)-based tools such as ClusterFinder [16] and antiSMASH. This improved the identification of BGCs in bacterial genomes and contributed to the discovery of novel BGCs encoding natural products that could not be detected by experimental methods.

Proteins can expand their functional capabilities by forming complexes with other proteins or ligands. As in other types of gene clusters, the formation of protein complexes plays a crucial role in BGCs. Notably, protein–protein interactions observed in carrier proteins [17], trans-type acyltransferase (AT) polyketide synthase (PKS) [18], cis-type AT PKS [19], type I/II PKS, and fatty acid synthase [20,21] have been extensively studied and discussed. These interactions are key components of the biosynthetic machinery. In homomultimer complexes, residues that are distant in the primary and tertiary structures assemble at the multimer interface to form a single binding pocket [22]. Additionally, some heteromeric complexes influence the stability, specificity, and catalytic activity [23,24]. Because these interactions underlie the remarkable structural and chemical diversity of the products, many enzymes in these systems have been structurally characterized to clarify their molecular basis to date. Nevertheless, most BGCs still harbor numerous proteins of unknown function, often annotated simply as “hypothetical protein” in MIBiG. Sequence-based bioinformatics tools generally cannot predict the function of these proteins unless homologous proteins with known functions have already been characterized. This limitation represents a major challenge for the comprehensive understanding of their biosynthetic pathways.

Highly accurate prediction of protein tertiary structures, as exemplified by AlphaFold2 (AF2) [25], has markedly advanced structural bioinformatics. Structures predicted with high confidence are often sufficiently accurate to be used as search models for the molecular replacement method in x-ray crystallography [26,27], as well as initial structures for molecular dynamics simulations and quantum mechanics/molecular mechanics calculations [28]. Furthermore, AlphaFold provides valuable predictions about protein–protein [29] interactions that are difficult to obtain from sequence-based bioinformatics tools alone. Notably, AlphaFold predicts inter-residue distances based on coevolutionary signals concealed in the multiple sequence alignments (MSAs) for the query sequence(s). When more than roughly 30 to 100 effective homologous sequences are available in the sequence database, this strategy enables accurate prediction without relying on experimentally determined homologous structures or prior knowledge of protein–protein interactions. One application of protein complex prediction is the discovery and experimental validation of the protein Tmem81, which is conserved across vertebrates during fertilization of sperm and egg but whose function could not be inferred prior to the study [30].

Here, we report a comprehensive search of all pairwise complex predictions among proteins encoded within each BGC in MIBiG using AlphaFold3 (AF3) [31]. These computational analyses enabled highly sensitive detection of potential homo- and heteromultimer formation that were previously difficult to predict using conventional sequence-based bioinformatics tools. This approach led to the identification of novel pairs of proteins that are likely to act cooperatively. Furthermore, our results suggest that numerous structurally homologous heterocomplexes exist in BGCs and that many of them involve proteins previously annotated as having unknown functions. Finally, we visualized the protein–protein interaction network maps to provide a comprehensive overview in the BGCs. Taken together, these findings provide important insights into previously uncharacterized proteins in BGCs, thereby providing valuable contributions to both experimental and computational researcher communities engaged in the investigation of secondary metabolites.

## Materials and Methods

### Experimental and technical design

**Fig. 1.**
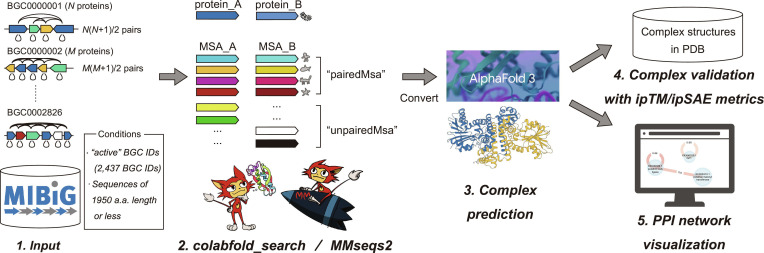
Computational workflow. (1) Input amino acid sequences. All pairs of identical or different protein sequences encoded within each BGC accession ID are enumerated, excluding those with a length more than 1,950 residues. (2) Generation of paired and unpaired MSAs for all the protein pairs are generated using MMseqs2 and *colabfold_search*. (3) Prediction of dimeric complexes for all protein pairs using AlphaFold3. The input JSON files are generated with *msatojson* subcommand implemented in our *alphafold3_tools*. (4) Validation of predicted structures using experimentally determined structures deposited in the PDB. The ipTM and ipSAE metrics are employed to assess the plausibility of the predicted complexes. (5) Visualization of protein–protein interactions (PPIs) that exceed a defined threshold in a web browser. Output JSON files containing ipTM and ipSAE metrics are also provided.

This study employed large-scale data curation, structure prediction, and validation of the predicted structures, along with network visualization. The overall workflow is illustrated in Fig. 1. All methods used were computational; however, for validation, we referred not only to structural information from the Protein Data Bank (PDB) but also to experimental interaction data. Detailed methodologies are described in the following sections.

### High-throughput structure prediction workflow

We selected the proteins derived from BGCs for comprehensive protein–protein complex prediction based on the following criteria. First, we focused on 2,437 “active” BGC accession IDs deposited in MIBiG version 4.0 (2024-11-15) that are currently maintained and updated. IDs marked as “retired” or “pending” were excluded from analysis. Second, among the proteins encoded in these BGCs, those with more than 1,950 amino acids, which are commonly found in PKS or nonribosomal peptide synthetases (NRPSs), were excluded from the prediction, because they can exceed the computational limits of modeling using AF3. The amino acid sequences were obtained from the GBK format files provided by MIBiG. Using the amino acid sequences that meet the criteria, we performed exhaustive predictions of homo- and heterodimeric complexes for each BGC.

### MSA generation and structure prediction

Despite recent advances, protein structure prediction with AF3 still relies on sufficient number of MSAs for the query sequences. To achieve both high accuracy and high-throughput prediction, we replaced the default MSA generation pipeline using HMMER 3 [32] implemented in AF3 with a faster alternative: the *colabfold_search* command in LocalColabFold [33]. MMseqs2 version 15-6f452 [34] was used to generate MSAs for the query sequences against the Uniref30_2302 and colabfold_envdb_202108 sequence databases (released on 2023 July 31), which can be downloaded from https://colabfold.mmseqs.com/. The obtained MSAs have been shown to be sufficient for producing highly accurate predicted models with AF2 or ColabFold in most cases [33]. In addition to unpaired MSAs, we also obtained paired MSAs to further improve the accuracy of complex modeling. The protein template search against the PDB100 database was not performed, as it is known to improve monomeric but not complex prediction accuracy. The MSA generation was performed on the SuperComputer Flow Type III subsystem at Nagoya University. This system comprises 16 Intel Xeon Platinum 8280M processors (2.7 GHz, 28 cores each) and 24 TiB of RAM per node. Each query took approximately 1 to 2 min on the calculation node. The obtained a3m-formatted MSA files were added to the “pairedMsa” and “unpairedMsa” keys of the AF3 input JavaScript Object Notation (JSON) file using the *msatojson* subcommand of the alphafold3_tools software that we developed and made available on our GitHub repository: https://github.com/cddlab/alphafold3_tools/. The “modelSeeds” value in the AF3 input JSON file was set to 1, and 5 models were generated for each prediction. For a dimer with a total number of residues below 1,500, the complex structure prediction was performed using the SuperComputer Flow Type II subsystem with NVIDIA GPU V100; for those with 1,500 to 2,000 residues, our in-house NVIDIA GPU RTX4090; and for those with 2,000 to 3,900 residues, NVIDIA GPU H100 (VRAM 94GB). The predicted aligned error (PAE) of the predicted structures was visualized using the *paeplot* subcommand of alphafold3_tools.

### Validation of the predicted complex structures

To identify biologically plausible protein–protein interactions in the AF3-predicted complexes, we employed 2 metrics: the interface predicted TM score (ipTM) and the interaction prediction score from aligned errors (ipSAE) [35]. The ipTM measures the accuracy of the predicted relative arrangement of subunits. Values ≥ 0.8 indicate confident and high-quality predictions, <0.6 suggest failure, and 0.6 to 0.8 represent an uncertain range [29,31]. The ipTM values are provided in the AF3 output JSON files. The ipSAE is a modified scoring function that evaluates the quality of predicted interactions by focusing on residue pairs likely to participate in the interface, rather than the entire sequence. We calculated ipSAE using our in-house implementation, the *metrics* subcommand, in the alphafold3_tools package. The subcommand also provides other interface evaluation metrics, including ipSAE_min [36], pDock [37], pDockQ2 [38], and LIS [39]. For the ipSAE calculations, both the PAE cutoff (pae_cutoff) and Cα-Cα distance cutoff (dist_cutoff) were set to 10. To assess the impact of MSA generation method on complex prediction, we compared the MMSeqs2/alphafold3_tools workflow with the HMMER3-based MSA generation implemented in the original AF3 pipeline. In the AF3 structure inference, the *max_template_date* parameter was set to 1900-01-01 to ensure that no template structures were used. Predictions were generated for all pairwise protein combinations from BGC0000001, BGC0000004, BGC0000006, and BGC0000007 (*n* = 1,184), and the resulting models were evaluated using ipTM, ipSAE, ipSAE_min, pDockQ, pDockQ2, and LIS.

We evaluated both ipTM and ipSAE metrics for the predicted homo- and heterodimeric structures by validating them against experimentally determined complexes. For proteins encoded in the BGCs listed in MIBiG version 4.0 and registered in the PDB, we retrieved their biologically relevant complex structures (also referred as “biological assemblies”) and stoichiometry information was retrieved from the PDB as of 2024 November 30. To minimize the impact of amino acid substitutions on protein oligomerization, the sequence identity threshold was set to 95%. Additionally, for complexes whose biological assemblies contained more than 2 chains and did not exceed 3,900 amino acid residues in total, we performed further structure predictions with the stoichiometries to obtain the corresponding ipTM and ipSAE metrics.

For the obtained heterodimeric structures, the root mean square deviation (RMSD) of the backbone heavy atoms (N, Cα, C, and O) between the 2 subunits was calculated using Gemmi version 0.7.3 (https://github.com/project-gemmi/gemmi) [40], with the maximum number of outlier rejection cycles set to 5. To prevent calculation errors caused by very short sequences (e.g., precursor peptides), RMSD values were not computed for proteins with fewer than 20 residues.

### Cloning and purification of recombinant proteins from *E. coli* BL21(DE3)

The rifampicin-resistant mutant TW-R50-13 chromosomal DNA was used as template DNA for polymerase chain reaction (PCR). Oligonucleotides used in this study were purchased from Fasmac Co., Ltd (Atsugi, Japan) and are listed in Ref. [41]. PCR was performed with KOD DNA polymerase (Toyobo, Osaka, Japan) or Expand High Fidelity PCR System (Roche Diagnostics) according to the manufacturer’s instruction. The amplified PCR products were then purified from 1% agarose gel using the Gel Extraction Kit (Qiagen, Tokyo, Japan). Purified PCR products were ligated into pBluescript II SK(+) vector or pT7Blue vector using restriction endonucleases (New England Biolabs, Ipswich, MA) and DNA ligase (Toyobo, Osaka, Japan). *Escherichia coli* DH5α was transformed with the resulting plasmids followed by purification with a GenElute Plasmid Miniprep Kit (Sigma-Aldrich) and outsourced for DNA sequencing (FASMAC, Tokyo).

pETDuet-1 vector (Novagen, Darmstadt, Germany) was used for the coexpression of 2 target genes in *E. coli* BL21(DE3) strain. pETDuet-1-*mbg7*/*mbg5* was constructed by cloning *mbg7* into the first multiple cloning sites (MCS1) of pETDuet-1 vector. Following the transformation of *E. coli* DH5α with the resulting construct and subsequent plasmid purification, *mbg5* was cloned into the second multiple cloning site (MCS2).

After transformation of *E. coli* BL21(DE3), each transformant was cultivated in 200 ml of TB medium (tryptone 1.2%, yeast extract 2.4%, glycerol 0.56%, K_2_HPO_4_ 1.25%, and KH_2_PO_4_ 0.23% supplemented with corresponding antibiotics) at 37 °C until OD_600_ reaches 0.4 to 0.6. After cooling the culture on ice for 10 min, isopropyl β-d-thiogalactopyranoside was added to the culture at a final concentration of 100 μM to induce gene expression, and the culture was further incubated at 18 °C for 12 h. The cells were harvested by centrifugation at 4 °C (5,000 rpm, 10 min) and washed with 20 ml of Wash buffer (20 mM Tris-HCl, pH 8.0, 300 mM NaCl, 20 mM imidazole-HCl, pH 8.0, and 20% glycerol), followed by centrifugation at 4 °C (5,000 rpm, 10 min). The washed pellet was resuspended in Wash buffer again and lysed by sonication on ice. Cell debris was removed by centrifugation at 4 °C (17,000 rpm, 20 min). A 2-ml slurry of Ni-NTA Superflow Resin (Qiagen, Tokyo, Japan) was added and incubated with the supernatant at 4 °C for 1 h. The resulting mixture was loaded on an Econo-Pac chromatography column (Bio-Rad, Hercules, CA), and resins were washed with 30 ml of Wash buffer and then eluted with 10 ml of Elution buffer (20 mM Tris-HCl, pH 8.0, 300 mM NaCl, 250 mM imidazole-HCl, pH 8.0, and 20% glycerol). Each purified protein was examined by sodium dodecyl sulfate–polyacrylamide gel electrophoresis.

### Network visualization

The protein–protein networks obtained for the 2,437 BGCs were drawn using NetworkX version 3.5. Each protein was represented as a node, and edges were drawn between heterodimers that showed ipTM > 0.55 in the AF3 structural prediction. Proteins that formed homodimers at the same threshold were represented with self-loop edges. Then, the network maps were output in SVG image format and made available on a web server. Additionally, the JSON files and Python codes used for generating these network maps will be published as supplementary files, allowing users to regenerate them in their own environments using arbitrary thresholds.

## Results

### High-throughput complex prediction pipeline

We developed a computational pipeline for the systematic prediction of protein–protein complexes encoded within BGCs (Fig. 1). The pipeline takes as input the GenBank (.gbk) files associated with BGC accession IDs registered in MIBiG. Users may also provide GenBank files generated by antiSMASH or FASTA-formatted protein sequence files for unregistered BGCs. Here, we focused on 2,437 BGCs that are labeled as “active” in MIBiG and targeted proteins with up to 1,950 amino acid residues. For each BGC, complexes were predicted for all possible protein combinations, including self-pairs. Specifically, for a BGC containing *N* qualifying proteins, we performed *N*(*N* − 1)/2 predictions for heteromeric pairs and *N* additional predictions for homomeric pairs, resulting in a total of *N*(*N* + 1)/2 predictions per BGC.

Highly accurate structure prediction using AF3 requires the generation of MSAs for the input sequences. However, comprehensive prediction using the AF3 Web server is not feasible due to its usage limits (currently 30 runs per day). Moreover, the official AF3 inference code available on GitHub is also impractical for the high-throughput workflows, mainly because the MSA generation process using HMMER 3 [32] is slow and cannot be efficiently parallelized. To address these issues, we used *colabfold_search* from LocalColabFold [33] in conjunction with MMseqs2 [34] to rapidly generate MSAs. The resultant MSAs included both unpaired MSAs for individual proteins and paired MSAs generated by concatenating sequences from the same organism. These were assigned to the “unpairedMsa” and “pairedMsa” keys, respectively, in the AF3 input JSON file. This conversion was performed using the *msatojson* subcommand, which is part of our *alphafold3_tools* toolkit. Using this pipeline, we generated MSAs in 1 to 2 min per complex on a server with 768 GB of RAM or more and efficiently distributed the structure inference tasks for a total of 487,828 protein pairs across multiple GPU-equipped computers.

We compared the complex structure predictions generated using MMSeqs2 and alphafold3_tools with those obtained using HMMER3 in the original AF3 pipeline across 1,184 protein–protein complexes identified from 4 BGCs. The resulting confidence metrics showed strong agreement between the 2 approaches. In particular, ipSAE, ipSAE_min, pDockQ2, and LIS exhibited correlation coefficients of 0.90 to 0.93, indicating that these metrics generally yielded comparable values regardless of the MSA generation method. Most of these metrics, unlike ipTM and pDockQ, produced values of 0.0 for a large proportion of the complexes. These cases largely overlapped with complexes that also received low ipTM or pDockQ scores. Based on these observations, we chose to focus on ipSAE, together with ipTM, as the primary confidence metrics in the present study.

**Fig. 2.**
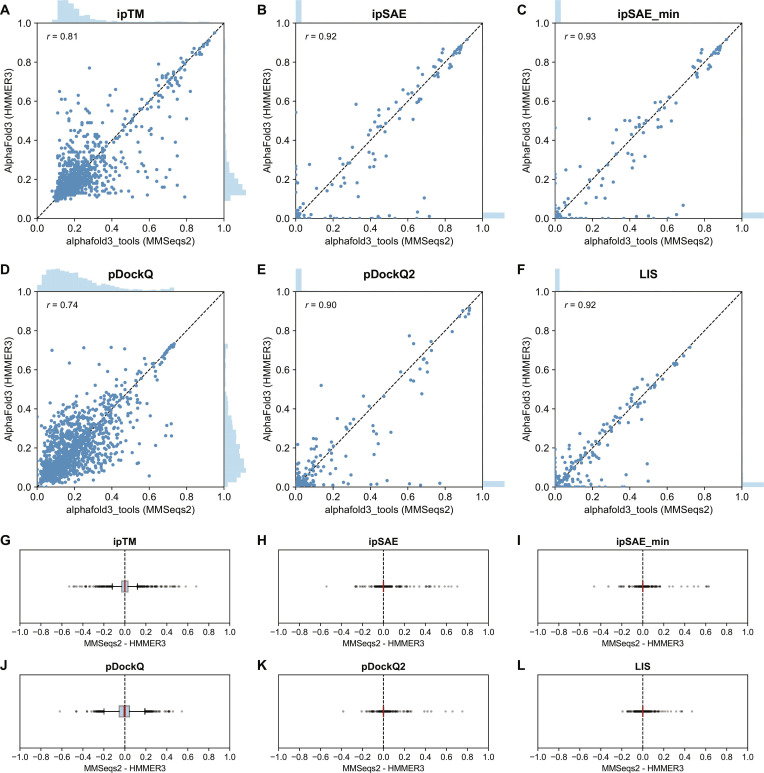
Comparison of prediction metrics obtained using MSAs generated by MMSeqs2/alphafold3_tools and HMMER3. (A to F) Scatter plots of ipTM (A), ipSAE (B), ipSAE_min (C), pDockQ (D), pDockQ2 (E), and LIS (F) calculated for structure predictions of 1,184 protein pairs. The *x*-axis represents values obtained using MSAs generated by MMSeqs2/alphafold3_tools, whereas the *y*-axis represents values obtained using MSAs generated by HMMER3. The dashed line indicates *y* = *x*. (G to L) Box plots showing the differences between metric values obtained using MMSeqs2/alphafold3_tools and HMMER3 for ipTM (G), ipSAE (H), ipSAE_min (I), pDockQ (J), pDockQ2 (K), and LIS (L). The box extends from the first quartile (Q1) to the third quartile (Q3), with the median indicated by a horizontal line. The whiskers extend to the most extreme data points within 1.5 × the interquartile range (IQR) from the box, and outliers beyond this range are shown as circles.

Interestingly, we observed several cases in which the predicted confidence metrics differed substantially between the MMSeqs2- and HMMER3-based MSA generation methods (Fig. 2). For example, for the interaction between AEK75492.1 and AEK75516.1 in BGC0000001, the MMSeqs2-based prediction yielded ipTM = 0.79 and ipSAE = 0.65, whereas the HMMER3-based prediction yielded ipTM = 0.11 and ipSAE = 0.0. In contrast, for the interaction between BAE71323.1 and BAE71335.1 in BGC0000004, MMSeqs2 yielded ipTM = 0.12 and ipSAE = 0.0, whereas HMMER3 yielded ipTM = 0.65 and ipSAE = 0.19. Across the 1,184 predictions, 71 protein pairs (6.0%) showed an absolute difference greater than 0.2 in ipTM between the 2 MSA generation methods, compared with 23 pairs (1.9%) in ipSAE. These observations suggest that the choice of MSA generation method can influence the predicted confidence of protein–protein interactions and may lead to either false negative or false positive predictions.

### Validation of the predicted complexes

We subsequently compared the predicted complexes with the experimentally determined protein complexes deposited in PDB to evaluate the validity. As of 2024 November 30, a total of 555 homomeric and 30 heteromeric protein entries were found in PDB to exhibit over 95% sequence identity with proteins registered in MIBiG ver. 4.0. Among the 555 homomeric proteins, 408 were annotated as homodimers, and 135 were annotated as homotrimers or higher-order assemblies that could be modeled on our GPUs. The remaining 12 entries could not be predicted due to their extremely large complex sizes. A high sequence similarity threshold of 95% was applied to minimize the effects of amino acid differences, insertions, or deletions that could alter the composition of residues at the multimerization interface and thereby lead to inaccurate measurements.

**Fig. 3.**
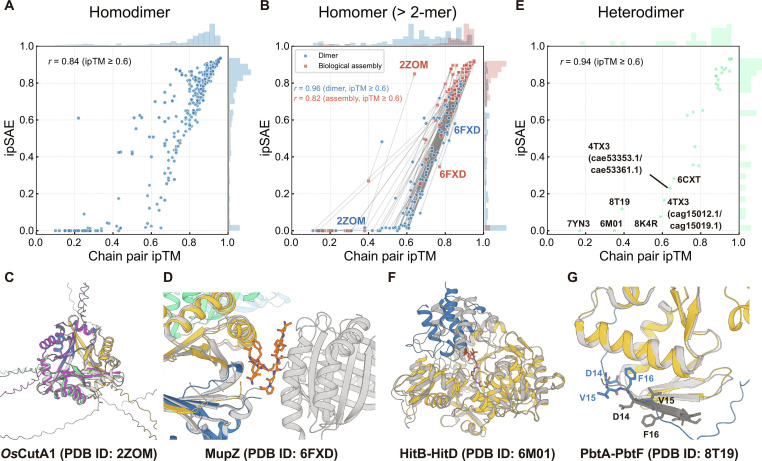
Validation of the predicted complexes. (A) Scatter plot of chain-level ipTM and ipSAE values for predicted homodimers (*n* = 408). Marginal histograms for each axis are displayed above and to the right. (B) Differences in ipTM and ipSAE between the predicted homodimer (blue) and the biological assembly (red). Only biological assemblies forming homomeric complexes larger than dimers and computable on our GPUs are included (*n* = 135). (C) Superimposition of the predicted *Os*CutA1 trimer (GenBank ID: BAT10611.1; blue, yellow, and green) and dimer (purple) with the crystal structure (PDB ID: 2ZOM). (D) Predicted MupZ homotetramer (colored) and its crystal structure (white). The artificial N-terminal peptide (orange) is shown in stick representation. (E) Scatter plot of ipTM and ipSAE values for predicted heterodimers (*n* = 30). (F) Predicted HitB (GenBank ID: BAR73008.1; yellow)–HitD (BAR73010.1; blue) and its crystal structure (white and gray). The cross-linking probe in the crystal structure is shown in brown stick representation. (G) Superimposition of the predicted PbtA (AGY49587.1; blue)–PbtF (AGY49592.1; yellow) complex with the crystal structure (PDB ID: 8T19; white and gray). Residues of PbtA at the interface are shown in stick representation.

For the protein structures registered in PDB as homodimers, our predicted dimeric models showed ipTM ≥ 0.8 for 294/408 (72.1%) and ipTM ≥ 0.6 for 358/408 (87.7%) (Fig. 3A). The ipSAE metric, which represents a corrected ipTM calculated using only interchain residue pairs with reliable PAE scores, highly correlated with ipTM above the 0.6 threshold, but approached zero below this cutoff (Fig. 3A and B). We next examined the 135 proteins annotated as forming higher-order homomeric complexes. When predicted as dimers, these proteins frequently yielded ipTM values around 0.6. However, when re-predicted with their stoichiometries of biological assemblies registered in PDB, the ipTM values substantially improved, exceeding 0.8 in most cases (Fig. 3B). A representative example is CutA1 from *Oryza sativa* (PDB ID: 2ZOM). In the dimeric prediction, the substructure was nearly identical to the crystal structure but exhibited low confidence scores (ipTM = 0.38; ipSAE = 0.001). In contrast, the trimeric prediction, corresponding to the biological assembly, closely reproduced the experimental structure, with improved ipTM and ipSAE values of 0.64 and 0.84, respectively (Fig. 3C). These results highlight the limitations of using ipTM or ipSAE thresholds for dimer prediction, especially when the intersubunit interface is small. Additionally, the ipTM and ipSAE metrics were further improved to 0.93 and 0.86, respectively, when its N-terminal long transit peptide region (residues 1 to 64) was not included in the prediction. This finding also indicated that the long-disordered region lowered ipTM (Fig. S1). Despite this, ipSAE remained high, underscoring its reliability as an evaluation metric in such cases.

Nevertheless, some of the predicted complexes did not exhibit high ipTM/ipSAE values even when using the stoichiometries of biological assemblies. Closer inspection revealed that several of these cases represented artifacts, either due to misassignments of stoichiometry inferred from the crystal packing using PDBePISA [42], or the inclusion of artificial sequence tags that induced unnatural oligomerization. The former example is MxaA in *Stigmatella aurantiaca* (GenBank ID: AAK57184.1; PDB ID: 4U7W), which is experimentally validated as a monomer [43]. The latter example is MupZ from *Pseudomonas fluorescens* (Fig. 3D; GenBank ID: AFD33556.1; PDB ID: 6FXD), which is registered as a homo-tetramer due to C-terminal His_6_-tag-induced tetramerization, although the native protein forms a dimer [44]. We summarized the proteins exhibiting low ipSAE and ipTM values in Table S1. These observations highlight the challenges of constructing fully reliable datasets of multimeric assemblies from PDB. Nonetheless, our findings suggest that an ipTM threshold of 0.6 is effective for screening proteins for the potential of homooligomerization.

### Validation of the predicted heteromeric complexes

For the 30 heteromeric complexes, we observed similar ipTM and ipSAE correlations to those in the homomeric complexes. Among these, 6M01 [45], 7YN3 [46], 8K4R [47], 8T19 (DOI: 10.2210/pdb8T19/pdb), and 4TX3 [48] showed ipTM < 0.6 (Fig. 3E). We further investigated the cause of these low ipTM values. First, in PDB entries of 7YN3, 8K4R, and 6M01 (HitB–HitD, Fig. 3F), a chemical reagent to induce covalent cross-linking between the 2 proteins was used to obtain the crystal structure of the heterodimeric complexes. Biologically, one of the proteins in these 2 PDB entries is an acyl carrier protein (ACP), which is known to form a transient complex with the other protein. Thus, it is speculated that the ipTM does not yield high values for such complexes. This may be because the stability of ACP-related complexes depends less on strong protein–protein interactions and more on the chemical properties of the substrate attached to the phosphopantetheine arm of ACP. ACPs function by transiently interacting with multiple partner enzymes to deliver and modify tethered intermediates during biosynthesis, rather than by forming stable protein complexes with a specific partner. Consistent with this property, relatively low ipTM values may reflect the weak protein–protein interactions in ACP complexes. In addition, the chemical structures of ACP-bound substrates are often unclear or unavailable in existing database and therefore could not be considered in this study. Combined with the current limitations in accurately predicting protein–ligand structures in AlphaFold3, these factors likely further complicate the accurate prediction of transient ACP complexes.

PDB ID 8T19 represents the complex structure of PbtA, a ribosomally synthesized and posttranslationally modified peptide (RiPP) precursor peptide, and its modifying enzyme PbtF from the GE2270 BGC (BGC0001155). The ipTM and ipSAE values were 0.39 and 0.1, respectively. Interestingly, AF3 predicted that the peptide binds to the β-strand region of PbtF; however, the predicted binding position was shifted by 2 residues compared with the crystal structure (Fig. 3G). We further investigated NisB from *Lactococcus lactis* subsp. *lactis* and StrB from *Streptococcus thermophilus* LMD-9 (BGC0001209) [49] to gain insights into proteins that bind RiPP precursor peptides. The crystal structure of NisB in complex with its precursor peptide NisA was not included in the validation dataset because these proteins were fused within the same chain (PDB ID: 4WD9) [50] or the peptide is chemically modified (PDB ID: 6M7Y) [51]. StrB shares 94.98% sequence identity with its homologous protein SuiB from *Streptococcus suis* (PDB ID: 5V1T) [52]. The predicted complex structures for both matched well with their corresponding crystal structures, showing no residue shift and high ipTM and ipSAE values (Fig. S2). However, because of the extremely limited number of available structures or experimental data on formation of RiPP precursor peptide–enzyme complexes, a statistical evaluation for them remains difficult.

Taken together, these results indicate that AF3 successfully predicted complex formation (ipTM ≥ 0.6) in 26 out of the 30 heteromeric cases. However, there are limitations in its predictive capabilities, particularly for transient complexes or those with small binding interfaces, such as those formed by ACPs or precursor peptides.

### Novel heterocomplexes

**Fig. 4.**
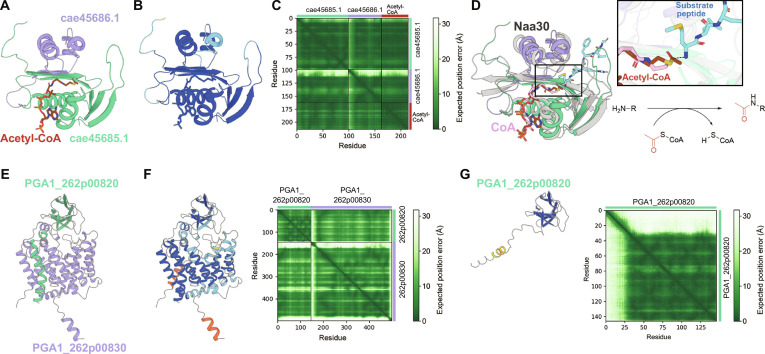
Examples of predicted complex models. (A) cae45685.1 (green)–cae45686.1 (purple)–Acetyl-CoA (brown) trimeric complex model. (B) The predicted complex colored by pLDDT (orange, 0 to 50; yellow, 50 to 70; cyan, 70 to 90; and blue, 90 to 100). (C) The predicted aligned error (PAE) matrix with chain coloring of panel (A) on the side bars. The dashed black lines indicate the chain boundaries. (D) Superimposition of the Naa30 crystal structure (white) on the predicted model shown in panel (A). The N-terminal region of major capsid protein as a substrate peptide (Uniprot ID: P32503) is shown as cyan stick. The proposed chemical reaction scheme for the complex enzyme using Acetyl-CoA is shown at the bottom right. (E) PGA1_262p00820 (green)–PGA1_262p00830 (purple) complex model found in BGC0000932. (F) pLDDT-colored predicted PGA1_262p00820–PGA1_262p00830 complex. The PAE matrix with chain coloring of panel (F) is shown on the right panel. (G) Predicted PGA1_262p00820 monomer structure colored by pLDDT. The right panel shows the PAE matrix. These data were obtained from the AlphaFold Protein Structure Database [88] (UniProt ID: I7E6A3).

Among 487,828 predicted protein pairs, 15,438 heterodimers exhibited ipTM ≥ 0.6, and 3,754 showed ipTM ≥ 0.8. These include complexes formed by previously uncharacterized proteins. Here, we can hypothesize the functions of these proteins based on the predicted models. For example, a heterodimer of cae45685.1 and cae45686.1 (Fig. 4A to C) found in borrelidin BGC from *Streptomyces parvulus* (MIBiG accession ID: BGC0000031) [53–55] is annotated as “hypothetical protein” in MIBiG, and to our knowledge, no studies have been reported for these proteins. BLASTp [56] search predicted both proteins have partial sequence similarity with GCN5-related *N*-acetyltransferase. Structure-based searches against the PDB100 database using the Foldseek webserver [57] indicated that the predicted complex structure resembles the single subunit Naa30 of the heterotrimeric NatC complex (PDB ID: 6YGD, chain A) [58], which catalyzes N-terminal acetylation (Nt-acetylation) of numerous eukaryotic proteins. Superimposition of the complex model on the Naa30 structure revealed that a binding pocket for the cofactor acetyl-CoA is formed between the cae45685.1–cae45686.1 interface, with the acetyl group of acetyl-CoA being exposed to the peptide-binding site of Naa30 (Fig. 4D). This suggests that this heterocomplex may also catalyze Nt-acetylation of its substrate. Recently, *Streptomyces mutabilis* sp. MII, which is thought to possess a BGC similar to BGC0000031, has been reported to produce *N*-acetylborrelidin B in addition to borrelidin (Fig. S3A) [59]. Given that borrelidin B, a known precursor of borrelidin, has an aminomethyl group at C12 carbon, it is plausible that the heterocomplex binds borrelidin B at a peptide-binding site analogous to that of Naa30 and catalyzes its acetylation to yield *N*-acetylborrelidin B (Fig. S3B and C).

We also identified several domain unknown function (DUF) proteins whose function appears to arise only upon complex formation. For example, PGA1_262p00820 (Genbank ID: AFO93364.1) in tropodithietic acid BGC from *Phaeobacter inhibens* DSM 17395 (MIBiG accession ID: BGC0000932) [60] was annotated as a DUF4399 domain-containing protein, a family for which functional characterization remains scarce. Our analysis predicted that this protein forms a complex with PGA1_262p00830 (AFO93365.1), a DoxX-family protein, with an ipTM of 0.82 and an ipSAE of 0.64 (Fig. 4E). Notably, the N-terminal region of PGA1_262p00820 was predicted to be disordered as a monomer; however, in the heterodimer, this region adopted an α-helical conformation with high pLDDT and formed an interaction interface with a helix of PGA1_262p00830 (Fig. 4F and G). Given that DoxX-family proteins are localized to the cell membrane, PGA1_262p00820 is expected to be embedded with PGA1_262p00830 in the cell membrane. Thus, the 2 proteins may act cooperatively as membrane-bound enzymes or transporters.

### Structurally homologous heterocomplexes

**Fig. 5.**
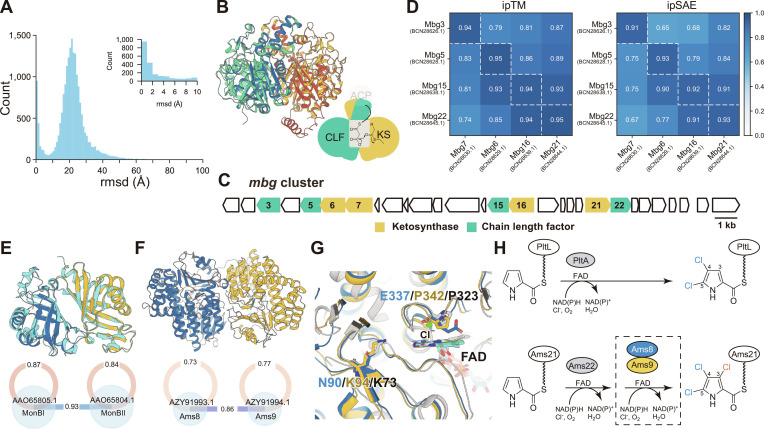
Predicted structurally homologous complexes. (A) Histogram of structural RMSD values between monomeric units of predicted heterodimer pairs. An inset shows a magnified view of the 0 to 10 Å range. (B) Superimposition of the predicted complex structure of actinorhodin polyketide β-ketoacyl synthase alpha (KS) and beta (CLF) subunits (blue and red) on the crystal structure (PDB ID: 1TQY; green and yellow). KS is shown in blue and green, and CLF is shown in red and yellow. (C) *mbg* cluster. Only the genes encoding KS and CLF are depicted. (D) Computed ipTM and ipSAE values for each complex prediction of the 4 KS (horizontal) and 4 CLF (vertical) subunits in *mbg* cluster. (E) Superimposition of the predicted MonBI (blue)–MonBII (yellow) heterodimer on the crystal structure of the MonBI homodimer (PDB ID: 3WMD; cyan). The computed ipTM values for each homo- and heterodimer are shown below. (F) Predicted Ams8 (blue)–Ams9 (yellow) complex. (G) Close-up view of the FAD binding site in the predicted structures of Ams8 (blue) and Ams9 (yellow). The pltA crystal structure (PDB ID: 5DBJ) is shown in gray. Residues presumed to be important for chlorination activity are shown in stick representation. (H) Proposed trichlorination mechanism in the armeniaspirol BGC (bottom). Ams8 and Ams9 may form a heterodimeric enzyme complex that catalyzes chlorination at the 3-position (dashed box). Formation of the dichloropyrrole moiety catalyzed by PltA during pyoluteorin biosynthesis is shown on the top for comparison.

By examining the predicted heterodimers, we identified 952 pairs (4.9%) in which the structural RMSD between constituent proteins was ≤ 1.0 Å, and 1,390 pairs (7.1%) with RMSD ≤ 2.0 Å (Fig. 5A). The median ipTM and ipSAE values for the protein pairs with RMSD ≤ 2.0 Å were 0.82 and 0.71, respectively, indicating that these predictions were made with high confidence. This observation suggests that structurally homologous heterodimers are present across BGCs and contribute to the biosynthesis. A well-characterized and popular example is a ketosynthase (KS)–chain length factor (CLF) complex, which constitutes the central catalytic machinery in Type II PKSs. In this complex, the KS subunit catalyzes the decarboxylation of the malonyl unit tethered to a phosphopantetheinyl arm of an ACP, and the condensation of the resulting enolate anion with a growing polyketide chain attached to its catalytic cysteine. The CLF subunit is a KS homolog lacking catalytic residues, but the internal tunnel formed by the complex plays a critical role in determining the polyketide chain length. The actinorhodin polyketide beta-ketoacyl synthase alpha (GenBank ID: CAC44200.1) and beta (CAC44201.1) subunits of BGC0000194 have been identified as KS and CLF, respectively, and their heterocomplex structure was determined (PDB ID: 1TQY) [61]. The protein pair was predicted to form a complex with high ipTM and ipSAE values of 0.96 and 0.93, respectively, and closely matched the crystal structure (Fig. 5B). Interestingly, despite the KS and CLF subunits sharing the same fold with an RMSD of 1.02 Å, the homodimeric prediction for KS yielded relatively low scores (ipTM = 0.65, ipSAE = 0.38), and CLF showed almost no signal (ipTM = 0.20, ipSAE = 0.0). These findings indicate that AF3 is capable of correctly identifying heterocomplex formation independent of overall structural similarity between monomers.

Subsequently, we examined whether ipTM and ipSAE could identify correct KS–CLF pairs within a BGC cluster encoding multiple KS and CLF proteins. In our previous work, we experimentally validated the correct pairing in *mbg* cluster (BGC0002441; Fig. 5C) as Mbg7–Mbg3, Mbg6–Mbg5, Mbg16–Mbg15, and Mbg21–Mbg22 [41]. Our all-by-all complex predictions for the 4 × 4 combinations showed that both ipTM and ipSAE values reached their maximum values for the experimentally validated pairs (Fig. 5D). Notably, ipSAE yielded lower values for incorrect pairings than ipTM, indicating greater discriminatory power. To further evaluate these predictions, we experimentally tested a subset of the *mbg* KS–CLF combinations by coexpressing His-tagged Mbg7 or Mbg6 with untagged Mbg3 or Mbg5 and assessing complex formation. Mbg7–Mbg3 (ipTM = 0.94, ipSAE = 0.91) and Mbg6–Mbg5 (ipTM = 0.95, ipSAE = 0.93) coeluted during Ni-NTA affinity purification, indicating strong interactions in solution (Fig. S4). In contrast, for the Mbg7–Mbg5 combination (ipTM = 0.83, ipSAE = 0.75), only Mbg7 was recovered in the eluate, indicating that the 2 proteins did not form a detectable complex under the tested conditions. These results demonstrate that false positive predictions can occur even when relatively high ipTM or ipSAE metrics are obtained. A similar trend was observed in the Ysf cluster (BGC0002547) [62], which encodes 3 KSs and 3 CLFs, where the highest values again corresponded to the experimentally confirmed pairings (Fig. S5). Collectively, these results demonstrate that the ipTM and ipSAE metrics provide reliable indicators for identifying likely KS–CLF pairs and predicting their released polyketide intermediates, even when the corresponding genes are not located adjacent within the BGC. However, the Mbg7–Mbg5 result also highlights that high metric values alone do not guarantee a true interaction and that experimental validation remains important for confirming predicted protein–protein interactions.

Our comprehensive predictions further identified 381 protein pairs that are likely to form both homodimers and heterodimers, meeting the criteria of ipTM ≥ 0.6 and ipSAE ≥ 0.6. A representative example is the pair MonBI and MonBII from the monensin BGC (BGC0000100). Although the crystal structure of the MonBI homodimer was reported in our previous study (PDB ID: 3WMD) [63], biological assays revealed that MonBI alone lacks the enzymatic activity but can form a heterodimer with MonBII, and this heterodimer is enzymatically active [63,64]. More recently, our group has determined the crystal structure of the MonBI–MonBII heterodimer (PDB ID: 9KW6) [64]. AF3 predictions indicated that MonBI and MonBII form homodimers with ipTM/ipSAE values of 0.87/0.83 and 0.84/0.71, respectively, while the predicted MonBI–MonBII heterodimer exhibited a higher ipTM/ipSAE value of 0.93/0.88 (Fig. 5E). The predicted heterodimer structure closely matched both the experimentally determined MonBI homodimer (RMSD = 0.74 Å) and the MonBI–MonBII heterodimer (RMSD = 0.89 Å). This result suggests that dimer formation between these 2 proteins is competitive, and that the heterodimer is preferentially formed.

Proteins predicted to form both homo- and heterodimers were also observed among other functional proteins. We highlight 3 flavin-dependent halogenase-like proteins—Ams8, Ams9, and Ams22—from the armeniaspirol BGC (BGC0002022) [65] as an example. Within the BGC, Ams21 and Ams22 share sequence similarities of 43% and 65% with PltL and PltA, respectively, from the characterized pyoluteorin BGC (BGC0000127), suggesting that Ams21 functions as an ACP and Ams22 as a flavin-dependent halogenase. Moreover, the structure of the active site of Ams22 is highly similar to that of PltA (PDB ID: 5DBJ) [66], which catalyzes the 4,5-dichlorination of the pyrrolyl moiety covalently attached to the phosphopantetheinyl arm of PltL, strongly suggesting that Ams22 catalyzes the same reaction (Fig. S6). However, unlike in the pyoluteorin BGC, the armeniaspirol BGC was reported to produce a 3,4,5-trichlorinated pyrollyl intermediate during the biosynthetic process [65]. Thus, the remaining halogenase-like enzymes, Ams8 and Ams9, are hypothesized to catalyze chlorination at the 3-position of the pyrrolyl moiety, although the mechanism remains unclear [65].

Our structure prediction pipeline showed that Ams8 and Ams9 may form homodimers with ipTM values of 0.77 and 0.73, respectively, whereas their heterodimer displayed a higher ipTM value of 0.86 (Fig. 5F). Interestingly, the predicted structure of Ams8 revealed that E337 is positioned at a site that would inhibit the binding of flavin adenine dinucleotide (FAD), an essential cofactor for halogenation. In addition, the conserved lysine residue required to mediate the reaction between the substrate and hypochlorous acid [67–69] generated from FAD, molecular oxygen, and chloride ions is replaced by N90 in Ams8 (Fig. 5G). These unusual residue substitutions strongly suggest that Ams8 lacks halogenase activity. By contrast, Ams9 and Ams22 retain these conserved residues. However, gene deletion of *ams8* completely abolished the biosynthetic process of armeniaspirol, whereas deletion of either *ams9* or *ams22* reduced its production by 100-fold but did not lead to complete loss [65]. This indicates that Ams8 is more essential for the biosynthetic pathway. Overall, these findings suggest that Ams9 functions as the enzyme catalyzing trichlorination of 4,5-dichloropyrrolyl–*S*-Ams22, while Ams8 acts as an auxiliary protein that supports the activity of Ams9 through complex formation (Fig. 5H).

### Visualization of the protein–protein interaction network map

**Fig. 6.**
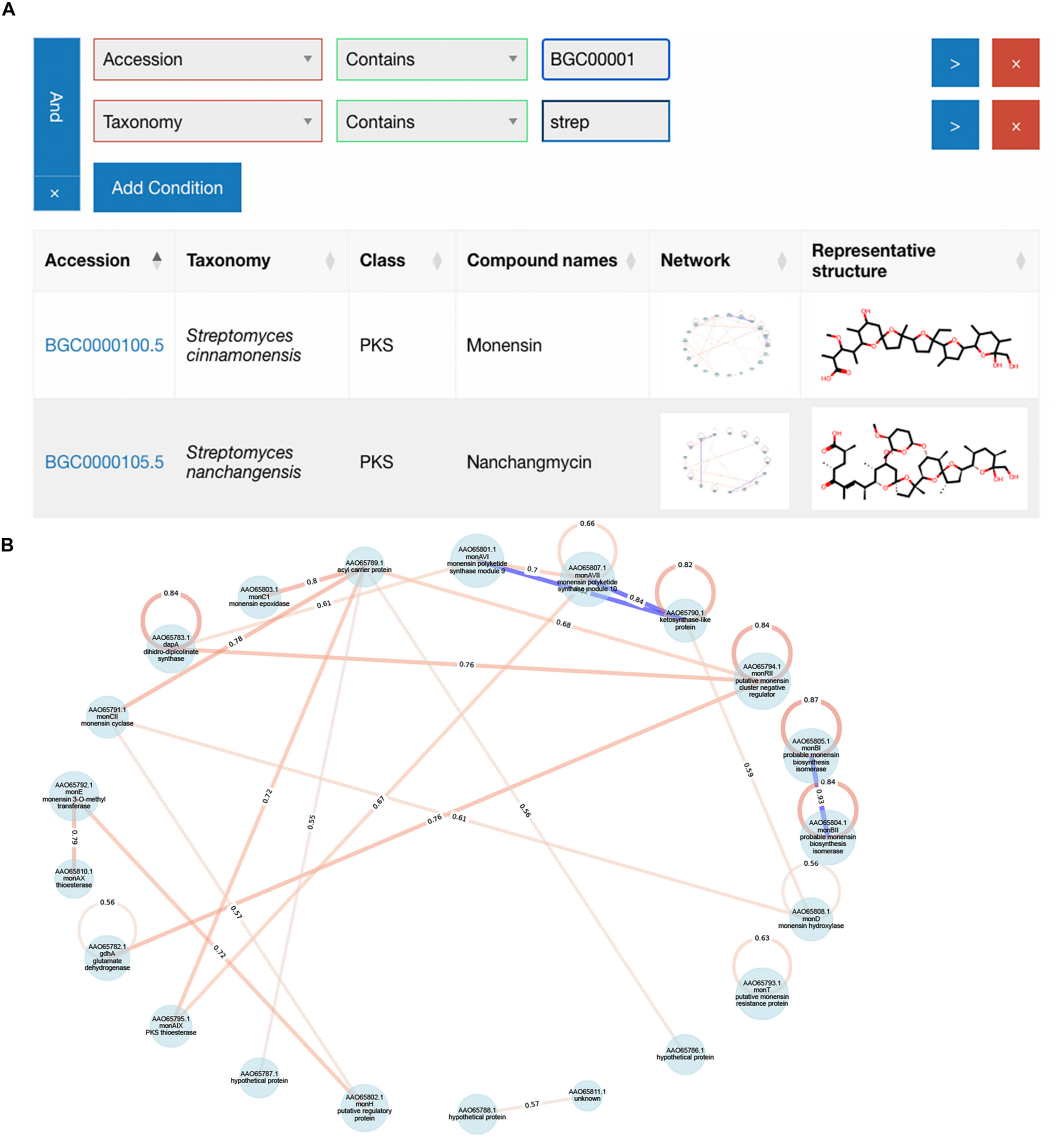
Visualization of the protein–protein interaction (PPI) network map. (A) The interface on the website. (B) The PPI network map in the monensin BGC (BGC0000100). The nodes represent proteins encoded within the BGC. Edges indicate predicted interactions, with labels showing the corresponding ipTM values. Blue edges denote structurally homologous interactions. Only nodes connected by edges above the ipTM threshold are displayed (ipTM > 0.55 was applied on the website).

To facilitate the use of our computational results by a broad range of researchers, we created a web site that visualizes protein–protein network diagrams for the 2,437 BGCs (http://vivace.bi.a.u-tokyo.ac.jp/network_in_bgc/publish.html). On this site, users can filter the data by MIBiG accession ID, taxonomy, class, or compound names, and view both the network diagrams and representative chemical structures of the products (Fig. 6A). Proteins are represented as nodes, and pairs of proteins predicted to form homodimers or heterodimers are connected by edges when they exceed a certain threshold (Fig. 6B). On this website, a relatively permissive threshold of ipTM > 0.55 is used to reduce the risk of overlooking weak but potentially meaningful interactions. To facilitate customized analyses, the JSON files and Python scripts used to generate the network diagrams have been deposited in Zenodo at https://doi.org/10.5281/zenodo.20536372. Users can therefore apply more stringent or more relaxed filtering criteria and prioritize interactions based on alternative metrics, including pDockQ, pDockQ2, LIS, and ipSAE_min.

## Discussion

One of the core challenges in the field of secondary metabolism and biosynthesis research is to elucidate the origins and biosynthetic mechanisms of the vast repertoire of natural products. In this study, we established a high-throughput complex prediction pipeline based on AF3 and demonstrated that it enables comprehensive detection of protein–protein interactions within BGCs. Our findings suggest that many proteins encoded within BGCs rely on homo- or heteromeric interactions for their biological functions, emphasizing the importance of protein complex formation in understanding biosynthetic pathways.

The high-throughput structure prediction of the present study is attributed to the use of MMSeqs2. The MMSeqs2 version 15 used in this study has been reported to achieve more than a 10-fold speed improvement over MSA generation approaches based on HHblits [70] and HMMER3 [33]. In addition, the recently released MMSeqs2-GPU (version 18) [71] performs this step on GPUs and achieves a 5.4-fold speedup compared with the CPU implementation of the same version. Consistent with these advances, the recently published “AlphaFast” [72] reported a 68.5-fold acceleration of the MSA generation stage in AF3 by incorporating MMSeqs2-GPU. Although MMSeqs2-GPU was not used in the present study, its adoption is expected to provide further computational gains, particularly for large-scale pairwise MSA generations. When predictions generated from different MSA construction methods were compared, ipTM and pDockQ showed high variability, especially in the low confidence range. In contrast, ipSAE, ipSAE_min, pDockQ2, and LIS, which are calculated using residues located at or near the predicted multimer interface, tended to converge toward to zero for low confidence predictions, resulting in agreement between the 2 MSA generation methods and higher correlation coefficients. However, it is noteworthy that the choice of MSA generation methods can have a substantial impact on the metrics in some cases.

Although our pipeline and network maps provide valuable insights into the interactome in BGCs, they have 3 limitations. First, AF2 (including AlphaFold-Multimer) and AF3 often fail to accurately predict weak or transient protein complexes. This limitation arises because their algorithms infer 3-dimensional protein structures primarily from coevolutionary signals in the MSA derived from individual amino acid sequence(s), and interchain interactions were not considered during their training processes [31]. Carrier proteins, such as ACPs in PKSs and peptidyl carrier proteins in NRPSs, exemplify this limitation. These proteins tether biosynthetic intermediates via a thioester bond to the thiol group of a phosphopantetheinyl arm attached to a conserved serine residue. They then interact with multiple catalytic domains in a specific yet transient manner to promote stepwise transformations of the substrate. Considering these biological properties, interactions between carrier proteins and their partner enzymes should be determined not primarily by protein–protein interactions, but rather by the chemical properties of the biosynthetic intermediates covalently attached to the carrier proteins, resembling protein–ligand recognition. In addition, because the predictions in this study did not include cofactors or biologically relevant substrates, any protein–protein interactions mediated or stabilized by these molecules could not be considered. Consequently, although identifying the partner enzymes of carrier proteins is crucial for studying biosynthetic pathways, accurate prediction of such interactions remains challenging. Similarly, predicting the binding position of precursor peptides in RiPPs is also difficult for the same reason.

Second, our computational pipeline did not perform complex predictions for proteins larger than 1,950 residues due to computational limitations. The number of proteins exceeding this length threshold is summarized in Table S2. Such proteins were rarely observed in ribosomal, terpene, and saccharide BGCs (0% to 1%), whereas they were more common in PKS- and NRPS-associated biosynthetic classes, where large synthases accounted for an average of 9% to 10% of the encoded proteins. These large proteins are typically type I PKSs or NRPSs, where multiple catalytic domains are linked within a single polypeptide chain to form the core scaffold of the product. While these modular proteins mainly assemble the product through their intramolecular domain–domain interactions, some tailoring enzymes within the same BGC can modify intermediates tethered to the modular proteins, thereby expanding the chemical diversity of the final products [73,74]. Prior prediction of these interactions could help identify novel tailoring enzymes responsible for introducing unusual functional groups; however, such predictions were not fully performed in this study.

Third, our method may fail to correctly predict the complex formation of proteins with potentially conformational changes. Although *talaromyolides* tlx BGC from *Talaromyces purpureogenus* has not been registered in the current MIBiG dataset yet, the TlxJ–TlxI heterodimer crystal structure (PDB ID: 7VBQ) [75] is a notable case to discuss the issue (Fig. S7A). While sequence analysis suggests that both TlxJ (GenBank ID: BDC03472.1) and TlxI (BDC03471.1) were nonheme iron oxygenases and the RMSD between the 2 proteins was 1.6 Å, TlxI is suggested to be enzymatically dysfunctional as oxidases due to the lack of an arginine residue for α-ketoglutarate binding and serves as an auxiliary protein to assist in TlxJ expression enhancing its catalytic activity [75]. The gel filtration analysis and the x-ray crystallography indicated that TlxI is monomeric (PDB ID: 7VBR) [75]. Thus, TlxI would be expected to show low ipTM/ipSAE values in the homodimeric prediction and high values in prediction with TlxJ. However, while TlxJ–TlxI was correctly predicted as a heterocomplex with an ipTM of 0.91 and an ipSAE of 0.86 (Fig. S7B), TlxI was incorrectly predicted to form a homodimer with a relatively high ipTM of 0.85 and an ipSAE of 0.75 (Fig. S7C). Interestingly, the crystal structures revealed that TlxI undergoes a large conformational change when forming a heterocomplex with TlxJ. Nevertheless, all the predicted TlxI structures closely resembled the conformation observed in the TlxJ–TlxI heterocomplex crystal structure (Fig. S7C and D). This observation is consistent with the caveat that AF3 does not adequately predict proteins that undergo conformational changes [31]. Because AF3 predominantly predicted the TlxJ-bound conformation of TlxI, the high structural similarity between TlxI and TlxJ likely resulted in a false-positive prediction of TlxI homodimerization. By contrast, as described in Results, our research group recently demonstrated that MonBI assists the folding of MonBII from an intrinsically disordered state into its correct 3-dimensional structure, thereby promoting preferential formation of the MonBI–MonBII heterodimer [64]. These experimental findings are consistent with the dimeric predictions in this study, in which the MonBI homodimer was predicted with an ipTM of 0.87 and an ipSAE of 0.71, the MonBII homodimer with an ipTM of 0.84 and an ipSAE of 0.83, and the MonBI–MonBII heterodimer with the highest values (ipTM = 0.93 and ipSAE = 0.88). Importantly, unlike the TlxJ–TlxI system, no substantial conformational change was observed for MonBI between the homodimeric (PDB ID: 3WMD) and heterodimeric (PDB ID: 9KW6) structures. Taken together, these observations suggest that for proteins capable of undergoing conformational changes (e.g., TlxI), AF3-based complex prediction may yield false positive even when high ipTM/ipSAE values are obtained. To our knowledge, MSA subsampling with AF2 or ColabFold [76] is one of the best strategies for predicting such conformational changes in advance. However, we did not apply this approach in this study because of its high computational cost.

Although several technical challenges remain in accurately predicting protein complexes, our comprehensive approach uncovered numerous plausible complexes hidden within BGCs. Notably, the structurally homologous heterodimers identified in this study may serve as intriguing targets for future research, particularly in the context of microbial evolution. According to the genome streamlining theory, natural selection in prokaryotes tends to eliminate genetic redundancy and nonessential sequences, leading to the evolution of highly compact and efficient genomes [77,78]. Thus, the structurally homologous complexes are expected to possess distinct functional roles such as regulation or assistance in protein expression. Examples of the former function have been reported in both homocomplexes [79,80] and heterocomplexes [75], and their characterization is relatively straightforward. In contrast, the latter function can only be uncovered through coexpression of a specific protein combination, and its underlying molecular mechanisms remain largely unexplored. Among the reported cases, MonBI–MonBII [63,64] and LstA–LstB [81] become functional only when coexpressed. Coexpression of LxmK and LxmY has been shown not only to improve stability but also to increase yield [82]. Interestingly, TalA from *Talaromyces pinophilus* LD-7 [83] and Lsd19 from *Streptomyces lasaliensis* [84–87] have been identified as naturally occurring chimeric enzymes, in which 2 structurally homologous protein domains are fused into a single polypeptide chain. Such protein (or domain) pairs may exist to expand the range of catalytic functions that can be achieved by a single enzyme alone.

The results of this study provide a comprehensive overview of the proteins encoded within BGCs and highlight protein complexes that represent promising targets for future investigation. By leveraging the relationship between protein structure and function, our approach enables the consideration of molecular interactions that cannot be explicitly inferred from sequence information alone. Our analyses suggest that enzymes previously regarded as missing pieces in biosynthetic pathways may, within some BGCs, depend on complex formation for stable and efficient function, as exemplified by the experimentally characterized MonBI–MonBII and TlxJ–TlxI complexes. Future studies combining predicted structures, interaction networks, and information on biosynthetic intermediates may enable the reconstruction of detailed biosynthetic pathways from the starter substrates to final products. In parallel, comparative analyses of BGCs across diverse organisms may offer insights into the evolutionary relationships and diversification of natural product biosynthesis. Collectively, these bottom-up, structure-guided approaches will advance our genetic and biochemical understanding of natural product biosynthesis and provide a foundation for rational engineering enzymes.

## Data Availability

The predicted protein–protein interaction network and JSON files underlying this article are available in Zenodo at https://doi.org/10.5281/zenodo.20536372. The Python scripts used for the preprocessing, evaluation, and visualization are publicly available at https://github.com/cddlab/bgccomplexbuilder. The *msatojson*, *paeplot*, *metrics*, and other utility subcommands to process the input and output files of AlphaFold3 can be downloaded as *alphafold3_tools* (https://github.com/cddlab/alphafold3_tools).

## Supplementary Materials

Supplementary 1 Figs. S1 to S7 Tables S1 and S2 References [89–96]
