# Supplementary material for: High-Throughput Prediction of Protein–Protein Interactions Uncovers Hidden Molecular Networks in Biosynthetic Gene Clusters
Source: Comput Struct Biotechnol J. 2026 Jun 29;35(1):0149. doi: 10.34133/csbj.0149 (PMC13311259; doi:10.34133/csbj.0149)
Supplement: Supplementary 1 — Figs. S1 to S7 Tables S1 and S2 References [89–96] [file csbj.0149.f1.zip › Supplementary Materials.docx]

Supplementary Materials

**
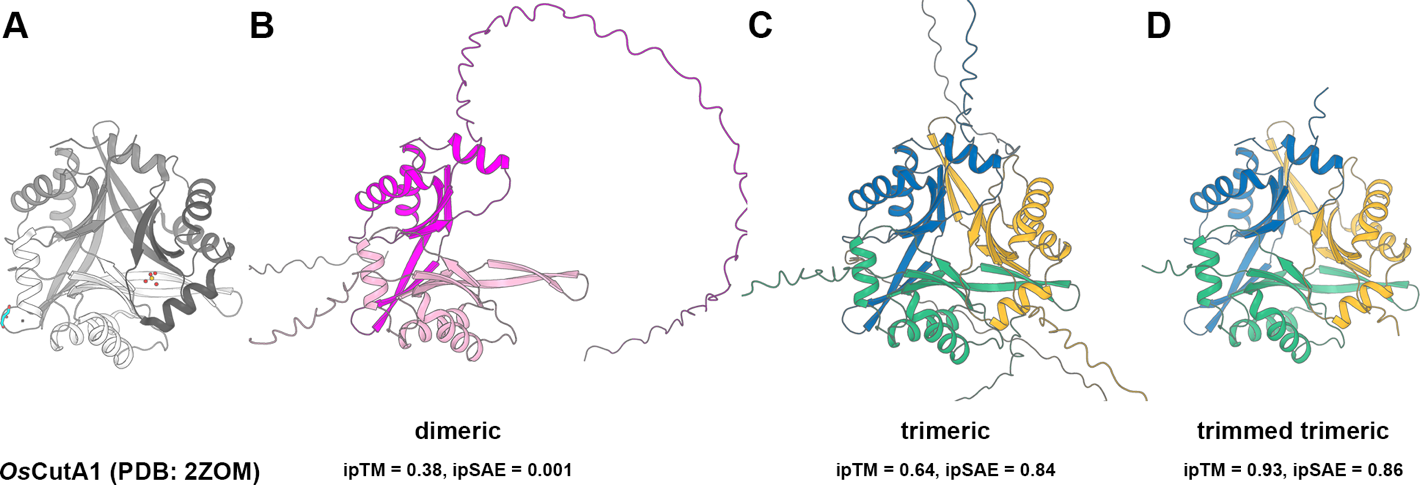
**

**Figure S1:** **Structural comparison of *Os*CutA1.** (A) Crystal structure (PDB ID: 2ZOM). (B) Predicted dimer structure. (C) Predicted trimer structure. (D) Predicted trimer structure using residues 65–177.

**
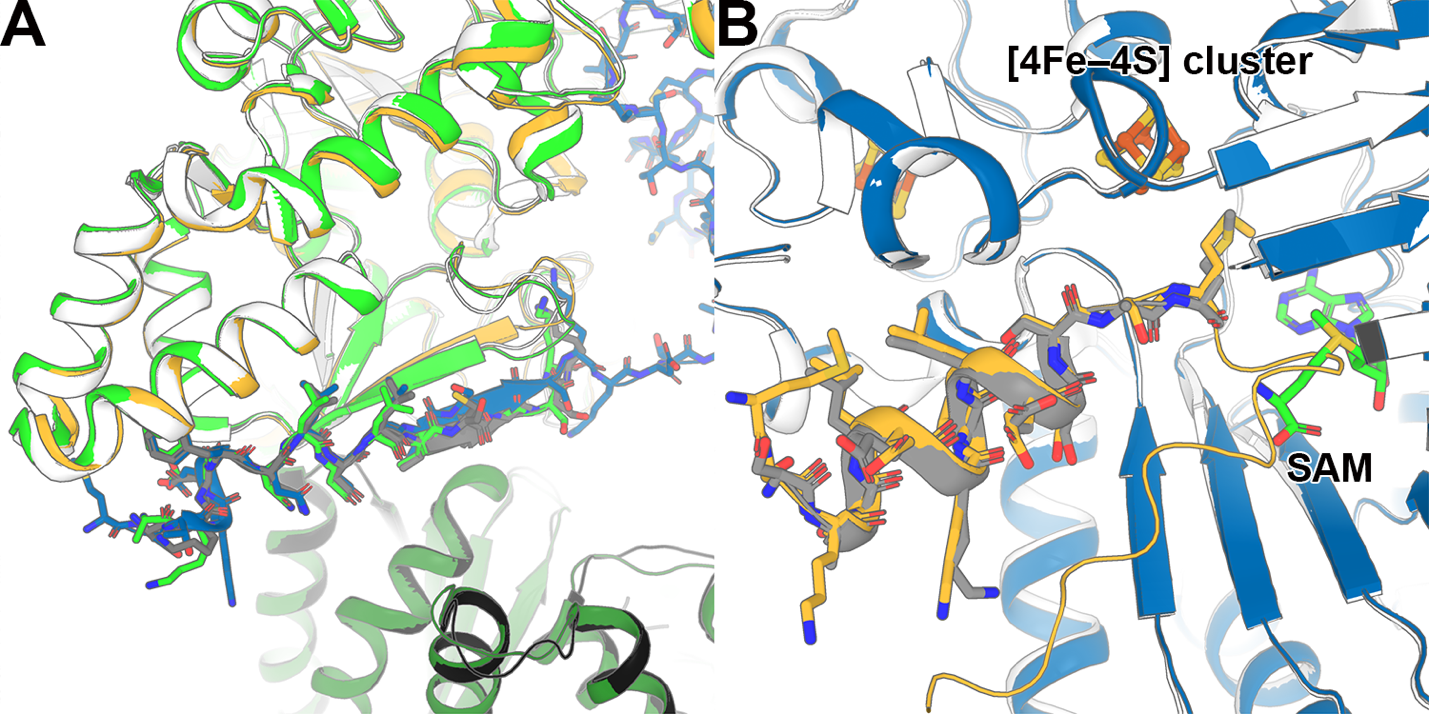
**

**Figure S2:** **Superimposition of predicted structures of RiPP precursor peptides and their tailoring enzyme complexes.** (A) Superimposition of the predicted NisA (blue)–NisB (yellow) model with the crystal structures (PDB IDs: 4WD9, green; and 6M7Y, white/gray/black). The predicted ipTM and ipSAE were 0.68 and 0.66, respectively. In 4WD9, the precursor peptide NisA is fused to NisB in the same chain. (B) Superimposition of the predicted StrA (orange)–StrB (blue) complex from *Streptococcus thermophilus* LMD-9 (BGC0001209) with the crystal structure of its homologous complex, SuiB (white)–SuiA (gray), from *Streptococcus suis* (PDB ID: 5V1T). The predicted ipTM and ipSAE were 0.82 and 0.79, respectively. SuiB and StrB share 94.98% sequence identity. The [4Fe–4S] cluster and (radical) SAM cofactor are shown as stick models.

**
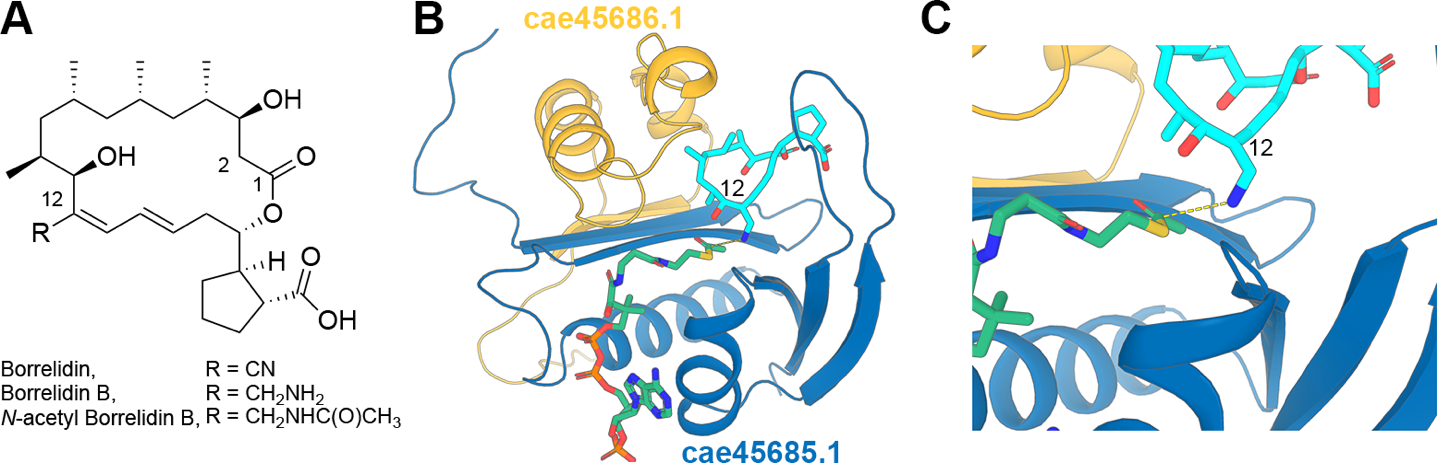
**

**Figure S3:** **Proposed catalytic mechanism of *N*-acetylborrelidin B.** (A) chemical structure of borrelidin, borrelidin B, and *N*-acetylborrelidin B. (B) Modeled cae45685.1 (blue)–cae45686.1 (yellow) heterodimer in complex with acetyl coenzyme A (green) and borrelidin B (cyan). (C) Close-up view of the two ligands. The dashed line is drawn between the putative reactive atoms.

**Figure S4:** **SDS-PAGE analysis of purified recombinant proteins.** SDS-PAGE (12 %) analysis of recombinant proteins. Each lane contains the Mbg7–Mbg5 (Lane I, calculated molecule weights (MWs): 46.3 kDa and 32.1 kDa), Mbg7–Mbg3 (Lane II, calculated MWs: 46.3 kDa and 38.6 kDa), Mbg6–Mbg5 (Lane III, calculated MWs: 41.5 kDa and 32.1kDa), Mbg7 (Lane IV, calculated 46.3 kDa), Mbg6 (Lane V, calculated 41.5 kDa). All these proteins were produced as N-terminally His-tagged proteins (marked with an asterisk) and purified by Ni-NTA affinity chromatography using Ni-NTA Superflow Resin. Lane M represents Marker.

**Figure S5**: Computed ipTM and ipSAE values for complex prediction of the KS (horizontal) and CLF (vertical) proteins in the same BGC. (A) kitacinnamycin A BGC (BGC0002109)[89,90]. (B) youssoufenes BGC (BGC0002547)[63]. The correct KS–CLF pairs identified by experiments are indicated by white dashed lines.

**Figure S6:** Comparison of PltA from the pyoluteorin BGC (BGC0000127) and Ams22 from the armeniaspirol BGC (GenBank ID: AZY92002.1). **a** Pairwise sequence alignment. Asterisks indicate residues located within 6 Å of the catalytic residue K73 of PltA. **b** Superimposition of the predicted structure of Ams22 (brown) onto the crystal structure of PltA (PDB ID: 5DBJ; white). Residues within 6 Å of the catalytic residue K73 of PltA are shown in stick representation. The modeled pyrrole substrate covalently attached to the phosphopantetheinyl arm is shown in yellow green. **c** Crystal structure of tryptophan 7-halogenase in complex with 7-chlorotryptophan (PDB ID: 2AR8)[91].


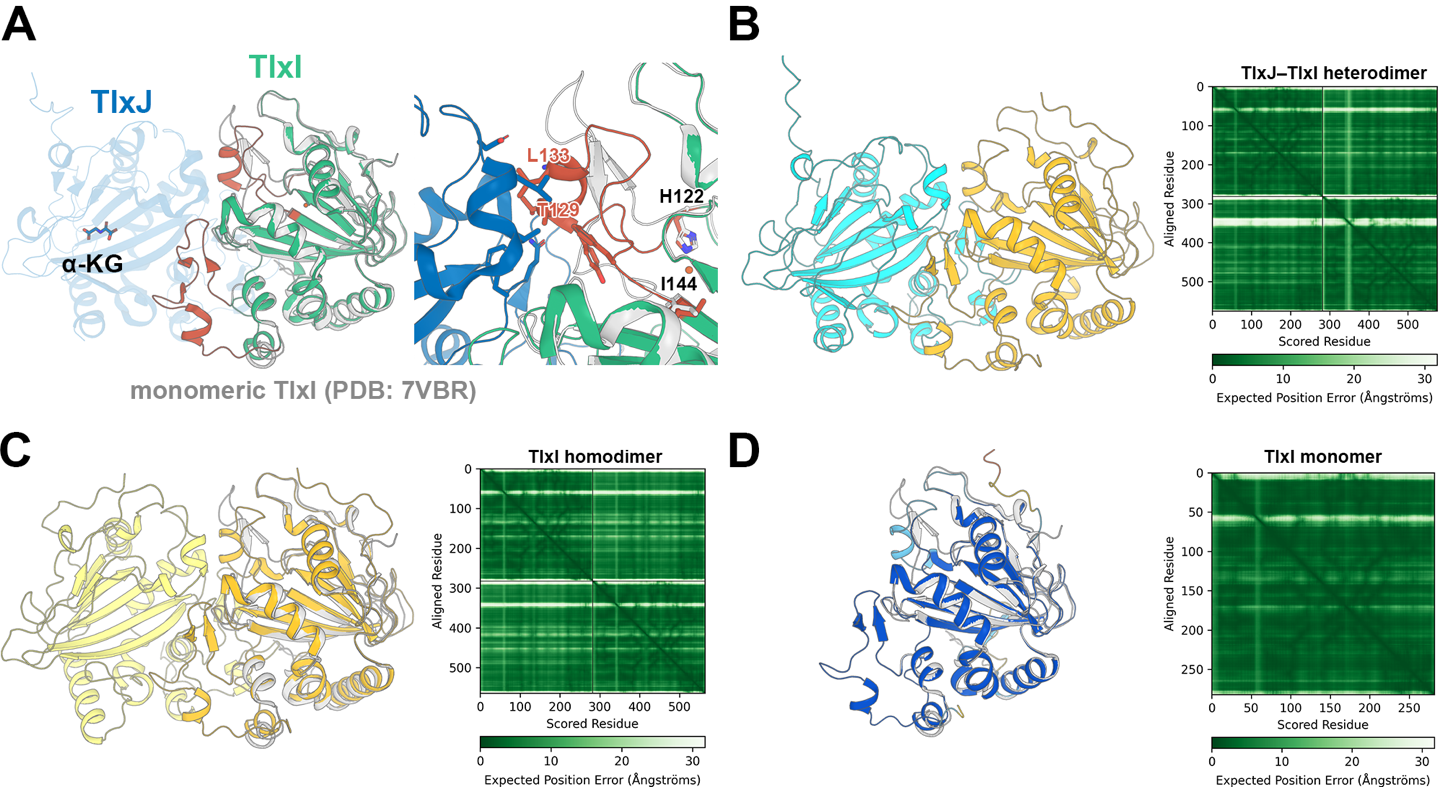


**Figure S7:** **Comparison of the crystal structure of TlxJ–TlxI and the predicted complex structure.** (A) Superimposition of the monomeric TlxI crystal structure (PDB ID: 7VBR) onto the heteromeric TlxJ–TlxI complex crystal structure (PDB ID: 7VBQ). Residues 122–144 of TlxI, which undergo large conformational changes, are highlighted in red. (B)–(D) Predicted models of the TlxJ–TlxI heterodimer (B), the TlxI homodimer (C), and the TlxI monomer (D). Their corresponding PAE matrices are shown in the right panels. In (C) and (D), the crystal structure of TlxI is displayed in white for comparison. In (D), TlxI is colored according to the pLDDT coloring (orange, 0–50; yellow, 50–70; cyan, 70–90; and blue, 90–100).

Table S1: Proteins exhibiting low ipTM and ipSAE values in our prediction pipeline.

| MIBiG accession ID | GenBank ID | PDB ID^a^ | Stoichio-metry registered in PDB | ipSAE (2-mer) | ipTM (2-mer) | ipSAE (biological assembly) | ipTM (biological assembly) | Notes |
| --- | --- | --- | --- | --- | --- | --- | --- | --- |
| 0000294 | BAC87903.1 | 6H7F, 6H7V, 6HCP | 3-mer,  monomer,  3-mer | 0 | 0.16 | 0 | 0.17 | Biophysical methods (gel filtration and light scattering) do not support a trimeric assembly.[92] |
| 0000610 | ACR48330.1 | 4ZA1 | 3-mer | 0.82 | 0.87 | 0 | 0.38 | Global Stoichiometry: Homo 3-mer, Local Stoichiometry: Homo 2-mer. |
| 0000668 | AHI58816.1 | 6GEM | 4-mer | 0 | 0.28 | 0 | 0.3 | Biological Assembly Evidence: gel filtration |
| 0000898 | AAC68683.1 | 8HZV, 8HZY | 4-mer, monomer | 0 | 0.23 | 0 | 0.29 | The C-terminal His6-tag of PDB ID: 8HZV may cause the multimerization. 8HZY is not. |
| 0000923 | AAC21672.1 | 3E59, 3EAT, 4YLM | Monomer,  3-mer  3-mer | 0 | 0.21 | 0 | 0.19 | Analyzed by size-exclusion chromatography and eluted midway between the expected times for a dimer and a trimer (data not shown).[93] |
| 0000929 | ABQ88342.1 | 5JDY, 5JDZ, 5JE0, 5JE1, 5JE2, 5JE3, 5JE4, 5JE5,  5JE6 | 4-mer | 0.012 | 0.38 | 0 | 0.2 | The authors conclude that the complexes are most likely to be monomeric.[94] |
| 0001738 | BBC43190.1 | 7DMN, 7DMO, 7E5T, 7E5U, 7E5V | 6-mer | 0 | 0.46 | 0 | 0.21 | Biological Assembly Evidence: gel filtration.[95] |
| 0002109 | QDJ74280.1 | 6J31, 6J32 | 3-mer | 0 | 0.15 | 0 | 0.16 | No description |
| 0002494 | AAD48879.1 | 1L5A | 3-mer | 0 | 0.13 | 0 | 0.14 | VibH is a monomer consisting of two domains.[96] |
| 0002681 | CAG44663.1 | 5NBC, 5NHK | 4-mer | 0.48 | 0.47 | 0.27 | 0.4 | Biological Assembly Evidence: light scattering, gel filtration, SAXS |

^a^ The underlined PDB ID was used to compute the ipSAE and ipTM metrics.

Table S2: Number and proportion of proteins exceeding 1,950 amino acids across major biosynthetic classes.

|  | PKS | NRPS | ribosomal | terpene | saccharide |
| --- | --- | --- | --- | --- | --- |
| No. of large proteins (>1950 a.a.) | 1913 | 1429 | 0 | 18 | 74 |
| No. of total proteins | 20635 | 14002 | 3058 | 1773 | 4538 |
| proportion | 0.093 | 0.102 | 0.000 | 0.010 | 0.016 |
